# Task Dependent Group Coupling and Territorial Behavior on Large Tiled Displays

**DOI:** 10.3389/frobt.2019.00128

**Published:** 2019-11-26

**Authors:** Anton Sigitov, André Hinkenjann, Ernst Kruijff, Oliver Staadt

**Affiliations:** ^1^Institute of Visual Computing, Bonn-Rhein-Sieg University of Applied Sciences, Sankt Augustin, Germany; ^2^Institute of Computer Science, University of Rostock, Rostock, Germany; ^3^School of Interactive Arts & Technology, Simon Fraser University, Vancouver, BC, Canada

**Keywords:** sensemaking, large display interaction, user study, group behavior analysis, immersive analytics

## Abstract

Large display environments are highly suitable for immersive analytics. They provide enough space for effective co-located collaboration and allow users to immerse themselves in the data. To provide the best setting—in terms of visualization and interaction—for the collaborative analysis of a real-world task, we have to understand the group dynamics during the work on large displays. Among other things, we have to study, what effects different task conditions will have on user behavior. In this paper, we investigated the effects of task conditions on group behavior regarding collaborative coupling and territoriality during co-located collaboration on a wall-sized display. For that, we designed two tasks: a task that resembles the information foraging loop and a task that resembles the connecting facts activity. Both tasks represent essential sub-processes of the sensemaking process in visual analytics and cause distinct space/display usage conditions. The information foraging activity requires the user to work with individual data elements to look into details. Here, the users predominantly occupy only a small portion of the display. In contrast, the connecting facts activity requires the user to work with the entire information space. Therefore, the user has to overview the entire display. We observed 12 groups for an average of 2 h each and gathered qualitative data and quantitative data in the form of surveys, field notes, video recordings, tracking data, and system logs. During data analysis, we focused specifically on participants' collaborative coupling (in particular, collaboration tightness, coupling styles, user roles, and task subdivision strategies) and territorial behavior. Our results both confirm and extend findings from the previous tabletop and wall-sized display studies. We could detect that participants tended to subdivide the task to approach it, in their opinion, in a more effective way, in parallel. We describe the subdivision strategies for both task conditions. We also detected and described multiple user roles, as well as a new coupling style that does not fit in either category: loosely or tightly. Moreover, we could observe a territory type that has not been mentioned previously in research. In our opinion, this territory type can affect the collaboration process of groups with more than two collaborators negatively. Finally, we investigated critical display regions in terms of ergonomics. We could detect that users perceived some regions as less comfortable for long-time work. The findings can be valuable for groupware interface design and development of group behavior models for analytical reasoning and decision making.

## 1. Introduction

Sensemaking is a mentally demanding process. It appears in many tasks, e.g., analytical (Bhavnani, 2002; Andrews et al., 2010) or incident and disaster management tasks (Wahlström et al., 2011). In general, the sensemaking process consists of two major loops of activities (Pirolli and Russell, 2011): an information foraging loop (Pirolli and Card, 1999) and a sensemaking loop (Russell et al., 1993). The information foraging loop includes such activities as seeking, filtering, reading, and extracting information. During this loop, the user works with small information portions at a time to gain knowledge about individual data items. The sensemaking loop includes activities such as connecting facts and building representations. During this loop, the user has to work with the entire data and has an overview of it. Thus, different loops require different approaches regarding visualization and interaction modalities, leading to different task conditions. In our study, we emulated the foraging loop in the *focus* task and the sensemaking loop in the *overview* task. During the focus task, participants had to process multiple documents; therefore, they had to seek for unprocessed documents, read documents, and solve tasks to extract information (document ID). During the overview task, participants had to connect documents using document IDs undergoing the connecting facts activity.

While working in groups, sensemaking becomes an even more complicated process. Social phenomena like collaborative coupling and territoriality emerge and accompany the entire process. For effective and efficient collaboration, an appropriate environment is of significant importance. That is where Immersive Analytics comes into play. Immersive Analytics investigates possibilities of immersing users in data employing new display and interaction technologies to foster the sensemaking process. One way to achieve an immersive experience is to place the user in a completely virtual environment, for instance, through a head-mounted display (HMD). HMDs, however, could not yet provide enough pixels and large enough field of view for large data visualization. Another way to immerse the user into data is to utilize a wall-sized high-resolution display. Due to inherent characteristics—namely large display real estate and the vast amount of pixels - large, high-resolution displays can cover the entire field of view of the user and provide the ability to scrutinize details within context (Marai et al., 2019). Thus, such displays are highly suitable for immersive analytics.

Marai et al. mentioned the following advantages of large, high-resolution displays in the sensemaking context: large display size and pixel density to show multiple representations simultaneously; ability to show context plus detail; enough space for group work (Marai et al., 2019). Systems using large, high-resolution displays often implement a whiteboard or tabletop metaphor with novel interaction techniques and devices to resemble well-known collaboration principles used in real-life communication (Guimbretière et al., 2001; Scott et al., 2003, 2004). Thus, they provide a more practical setting for co-located computer-supported collaboration in comparison to conventional desktop computer systems. Additionally, such displays can often visualize a significant amount of data and allow users to immerse themselves in the data. Moreover, large, high-resolution displays allow users to establish correspondences between their spatial position and orientation, and data elements on display (e.g., “I will see the document if I turn my head to the left”). As a result, users can use virtual and physical landmarks for objects finding (e.g., “The document is next to the chair” or “The document is further to the right from this one”). Hence, more useful and intuitive physical navigation can replace virtual navigation (Ball et al., 2007). Due to the many advantages of large, high-resolution displays, many researchers consider it pertinent to study the sensemaking process at them.

Collaborative sensemaking allows for looking at the problem from different perspectives and can profit from shared engagement and more qualitative communication in terms of subtle physical cues (Waltz, 2003). Moreover, researchers demonstrated the effectiveness of collaborative sensemaking in the context of real-world examples. For instance, exploration of ice-covered Lake Bonney (Marai et al., 2019), analysis of large-scale cosmological simulation data (Hanula et al., 2019), intelligence analysis (Vogt et al., 2011). To unfold the potential of large, high-resolution displays in the context of co-located collaborative sensemaking, we must provide appropriate user interfaces. For that, however, we first have to understand the specifics of group work that among others, entails collaborative coupling and territoriality phenomena. Collaborative coupling, in general terms, indicates the intensity of user-user interaction for accomplishing a task. Previous work mostly categorizes collaborative coupling into loosely coupled and tightly coupled (Gutwin and Greenberg, 1998, 2002; Tang et al., 2006; Isenberg et al., 2012). Sigitov et al. also investigated transitions between loosely and tightly coupled work for group model building (Sigitov et al., 2018a). Territoriality, on the other hand, addresses users' behavior that leads to the emergence of different regions with specific semantics and content (Scott et al., 2004).

Some related studies have focused on the user and group behavior during collaborative work around tabletops (e.g., Scott et al., 2004; Tang et al., 2006; Wallace et al., 2013). However, tabletop-based environments are different from vertical display environments. Tabletops' size is usually smaller since it is hard to utilize a large tabletop's center area. Additionally, users generally look down and not forward and may even have fixed seating places, which restrict physical navigation. These and other differences might impact collaborative coupling and territoriality. As such, designers of interactive spaces for vertically oriented displays can highly benefit from further investigation.

Furthermore, a limited body of work exists that has focused on co-located collaboration in front of high-resolution, vertical displays (e.g., Azad et al., 2012; Jakobsen and HornbÆk, 2014; von Zadow et al., 2016; Wallace et al., 2016). Some of these studies, however, used a different context as our study (e.g., public displays, gaming), and some did not allow for extensive physical navigation (Vogt et al., 2011; Jakobsen and HornbÆk, 2014).

In this work, we focused on collaborative coupling and territorial behavior of groups during two task conditions typical for sensemaking process, while working on a wall-sized display. Our study is similar to some extent to the previously conducted studies (e.g., Jakobsen and HornbÆk, 2014). However, it has three major differences:

- In contrast to other studies, we controlled the task conditions, which means that we let the participants to work in the focus task condition first and only after that they have completed the task of that condition, we let them move to the overview task condition. That provided us with an opportunity to get a clearer picture of group behavior during specific conditions.
- Next, we utilized mobile devices for interaction. That allowed users to move freely in front of the display interacting from any position within a defined area.
- We made use of fixed-position data disallowing the participants to move data assets. Fixed-position data is an important component of applications that work with spatial data. Spatial data–data where the position of individual data elements, as well as their shape and size, have a meaning – is a part of many group activities, like network analysis, route creation, interior/exterior design, disaster planning. Typical data examples within the mentioned application scenarios are city maps, floor plans, and weather data (Tang et al., 2006). Use of fixed-position data allowed us, on the one hand, to investigate users' attitude toward critical display regions. On the other hand, we could observe how users handle territoriality being disabled to shape territories employing assets grouping.

## 2. Related Work

In this section, we provide a brief overview of the most relevant related work on collaborative coupling and territoriality, the two main aspects we focused on in our user study.

### 2.1. Collaborative Coupling

Collaborative coupling describes the process of user-user interaction for task accomplishment. Researchers describe it in terms of collaboration tightness, coupling styles, user roles, and task subdivision strategies. In general, researchers subdivide collaborative coupling into two ranges: tightly and loosely (Gutwin and Greenberg, 1998; Scott et al., 2003; Morris et al., 2004; Tse et al., 2004). Within these, the intensity level may vary depending on a coupling style. Originally, tightly coupled work was defined as work that barely could take place without user-user interaction, while loosely coupled work describes rather a workflow where users act independently (e.g., Salvador et al., 1996; Gutwin and Greenberg, 1998, 2002).

Tang et al. adjusted the term collaborative coupling as “the manner in which collaborators are involved and occupied with each other's work” to highlight social aspects of the phenomena (Tang et al., 2006). They conducted two observational studies in the context of the collaborative exploration of fixed spatial data around tabletops. Analyzing the results, they could detect and describe six coupling styles the groups used during their work (e.g., same problem, same area).

Following, Isenberg et al. conducted another exploratory study around a tabletop system, where participants had to solve the VAST 2006 Challenge involving 240 documents (Isenberg et al., 2012). Opposite to the Tang et al. study, data was not fixed-position but represented through a set of floating document windows. The study revealed eight different coupling styles that were described based on participants' data view and personal interactions. The results overlapped at some points with those obtained in the study by Tang et al., while revealing four new styles.

A limited body of work exists that has focused on co-located collaboration in front of high-resolution, vertical displays. Jakobsen et al. recreated the exploratory study of Isenberg et al. using a multitouch wall-sized display (Jakobsen and HornbÆk, 2014). Again, the data was not fixed-position. Additionally, participants were forced to work next to the display because of touch input based interaction techniques. In contrast to the studies by Tang et al. and Isenberg et al., Jakobsen et al. used two different codes to describe coupling: one for visual attention and one for verbal communication. In total, they found five patterns of visual attention (e.g., same area - A and B looking at the same area) that in combination with verbal communication patterns could be used to describe coupling styles detected previously.

Liu et al. used different collaborative coupling styles to investigate a shared interaction technique for data manipulation on a wall-sized display (Liu et al., 2016). However, the system forced the participants to work in a particular manner. For instance, in conditions with shared interaction techniques, the participants were not able to solve the task individually. Thus, they could not work loosely coupled. Such restrictions disallow to observe natural behavior.

Rogers and Lindley investigated group behavior around both vertical and horizontal interactive displays (Rogers and Lindley, 2004). They observed that in the vertical display scenario, the participants frequently transitioned to loosely-coupled work in comparison to the horizontal display scenario. They could also observe the interactor user role (the person who interacts with the system). However, they utilized a relatively small vertical display (96 by 96 cm) and only one input device per group.

Vogt et al. investigated group behavior during collaborative sensemaking on a large, high-resolution display (Vogt et al., 2011). They described group behaviors concerning activities (e.g., extract and cluster) and user roles. In total, they identified two user roles: sensemaker and forager. Similar to our study, a curved display was used to let participants view the display in their peripheral vision. In contrast to our study, the provided interaction devices tethered participants to a particular place in front of the display.

The mentioned studies are most extensive in the domain of co-located collaborative coupling. More research exists that focused on co-located collaboration in front of high-resolution, vertical displays (e.g., Azad et al., 2012; Jakobsen and HornbÆk, 2014; von Zadow et al., 2016; Wallace et al., 2016). Some of these studies, however, used different context, e.g., public displays (Azad et al., 2012), gaming (von Zadow et al., 2016) or different input techniques (Jakobsen and HornbÆk, 2014).

### 2.2. Territoriality

Human territoriality is a social phenomenon that appears to influence interaction and communication processes during computer-supported cooperative work (CSCW). Sack has defined human territoriality as: “…* the attempt to affect, influence, or control actions and interactions (of people, things, and relationships) by asserting and attempting to enforce control over a geographic area*” (Sack, 1981). Territories can vary in scale (Sack, 1983; Taylor and Taylor, 1989) (e.g., from seats to cities) and can be controlled or claimed either by a single individual or by a group of persons (territory sharing) (Sack, 1983).

Tang conducted one of the first studies on territoriality in the context of CSCW (Tang, 1991). Two territory types were detected: *group* and *personal*. Later, Scott et al. conducted an extensive study within a non-digital tabletop environment to gain a deeper understanding of territoriality (Scott et al., 2004). As a result, they detected a new territory type: *storage territory*. Additionally, they described in detail characteristics (e.g., owners, position relative to users) of individual territory types. Many other researchers investigated territoriality in tabletop environments (e.g., Tang et al., 2006; Wallace et al., 2013), and introduced some interactions techniques (e.g., Scott et al., 2005; Moellers et al., 2011) to support this concept.

The is significantly less research on territoriality in the context of vertical displays. Azad et al. investigated territoriality on public wall-sized displays (Azad et al., 2012). In addition to known territory types, *unused territory*–display regions avoided by the user—was detected as a separate territory type. Jakobsen et al. observed territoriality on a large, vertical display (Jakobsen and HornbÆk, 2014). They noted that participants frequently worked in parallel without negotiating for space and shared the display evenly. They also stated that territories are more critical for loosely coupled than for tightly coupled work. Wallace et al. conducted an empirical study where they explored users' personal spaces around large public displays and confirmed the emergence of different territory types (Wallace et al., 2016).

## 3. User Study

We implemented two tasks to observe group behavior during collaboration on a wall-sized display. We used the Unity game engine and the Unity plugin for tiled displays (Sigitov et al., 2015, 2016). The tasks were carefully designed based on the sensemaking tasks used by (Andrews et al., 2010; Vogt et al., 2011; Isenberg et al., 2012; Jakobsen and HornbÆk, 2014). We extracted user-system interaction patterns and made the analytics part easier. We eased the analytics part for two reasons: time and participants. Our pilot studies showed that adding more documents (e.g., we started with 280 documents) or making the question more complex results in increased time for task accomplishment. For example, to merely open and close 280 documents without reading required over than 30 min. If we accumulate time needed for reading, understanding, and solving of a quiz question, it would take more than 2 h for the first task only. On the other hand, we observed that the strategy the participants used by task approach crystallized after 5–10 min from the beginning of the task, so there was no reason to make the tasks too long.

Another reason for easing the analytics part was that we aimed to be domain-agnostic. Having a domain in the experiment requires a large number of domain experts to experiment. We aimed to keep the participants motivated throughout the experiment by replacing domain texts with quiz questions.

In this study, we pursued abstract tasks, as commonly done in HCI (e.g., Pinelle et al., 2009; Bi et al., 2010; Liu et al., 2017). Our tasks relate in terms of user-system interaction processes to many real-world tasks, for instance, data exploration and sensemaking (Grinstein et al., 2006; Isenberg et al., 2012), data classification and sorting (Liu et al., 2014, 2016), route construction (Tang et al., 2006). Looking into all these tasks, one will find the same recurrent, canonical sub-processes:

- Target identification - decide what target to approach first/next.
- Target selection - indicate the target for the system.
- Target understanding - learn and understand the content and properties of the target.
- Sensemaking - conclude the relevance/significance of the target.

### 3.1. Focus Task

The focus task resembled the information foraging process. This process is an integral part of a typical visual analytics task that involves the processing of many documents (e.g., Grinstein et al., 2006; Andrews et al., 2010; Isenberg et al., 2012). The documents in our task had fixed positions on the display, which is a typical scenario for applications with spatial data, e.g., map-based applications. Use cases for such a scenario might include situations, where analysts have to investigate a series of events at specific geographic locations, e.g., identification of the best location for a new store in a particular region.

The task contained 70 processed, non-interactive documents, and 70 documents with questions. The documents had fixed positions; thus, participants could not move them. The questions were from the mathematics domain (e.g., *What is 884 divided by 26? What is one-third of 102? What is 24624 divided by 6?*) and physics domain (e.g., *What does supersonic speed exceed? What in Physics is the opposite of condensation? Which electronic components have impedance?*). Mathematical questions required a medium to a high level of concentration. We expected that any person with high-school level math skills would be able to answer these questions. In contrast, many physics questions required advanced skills. Combining these two types of questions, we expected to promote transitions between different coupling styles: for example, lack of knowledge in the physics domain should push participants toward tightly coupled collaboration, while mental arithmetic should instead dissolve tightly coupled collaboration. The full list of used questions can be found in the Supplementary Material.

#### 3.1.1. Procedure

During the task, the participants had to process 70 documents. Each document contained a question and four possible answers. The system marked a document as processed when the participant provided an answer to the contained question. Processed documents could not be re-opened and re-answered. The document remained unprocessed if the participant closed it without providing an answer. The system considered the task as accomplished if the participants processed all documents. There was no time constraint, and the task ended as soon as the participants answered all questions. The system notified the participants of task completion through a background color change. It was up to the participants to decide how they approached the task (e.g., divide documents and process them individually, or process all questions mutually), as we did not put constraints in this regard.

To process a document, the participant had to decide first what document she wants to process (*target identification*). Next, the participant must indicate the document for the system (*target selection*) by placing the pointer over the folder symbol and performing the tap gesture. Subsequently, the participant had to read and understand the question (*target understanding*). Finally, the participant had to provide an answer to reveal the document' ID.

#### 3.1.2. Visual Representation

**Figure 1:**
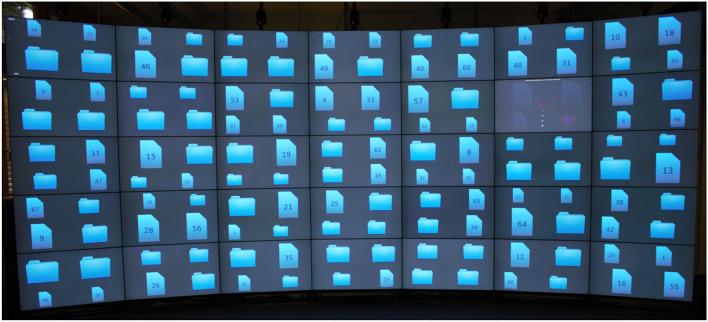
Focus Task: 140 symbols of folders and documents representing unprocessed and processed questions. The window in the top right corner shows a question with proposed answers.

At the beginning of the task, the display contained 70 processed documents and 70 unprocessed documents. The folder symbols represented unprocessed documents (“document is in the folder”-metaphor). The document symbols with an ID represented processed documents (“I took the document out of the folder”-metaphor). The symbols varied in size and had fixed positions on the display. Each display unit contained four symbols. The system placed the symbols in a way that no bezels occluded any symbol. Each display unit could contain only one opened document as it filled the entire display unit. Figure 1 shows a visual representation of the focus task with an open document.

#### 3.1.3. Interaction

Each participant had a virtual cursor. The participants controlled the cursors using the swipe gesture on the provided smartphones. To open a document, the participant had to place the cursor over the document and execute the tap gesture. With an opened document, the participant could not control the cursor. Instead, the participant could activate an option. Four of the options contained answers to the question. The fifth option was to close the document without providing an answer. To activate an option, the participant had to highlight it using the swipe gesture, subsequently executing the tap gesture.

#### 3.1.4. Motivating User

Although the IDs on the document symbols were only relevant for the overview task, we decided to utilize them to motivate the participants not to guess too much. The participants got instructions that if they would provide a wrong answer to a question, the showed ID on the document would be wrong as well. As a result, the assessment of the overview task would be worse, since the IDs serve as indicators for how the documents should be connected.

### 3.2. Overview Task

The overview task resembled a connecting facts activity. The activity is applicable, for instance, to connect visually similar home burglaries to visualize burglars' movements. If we look only at the interaction component of the activity, which is the subsequent execution of action at two different positions on a display, then the activity is directly comparable with any classification or sorting task (e.g., Liu et al., 2016). In the context of fixed-position data, this activity might be a part of a build a graph task, backtracking of a series of events task, or a route creation task.

#### 3.2.1. Procedure

**Figure 2:**
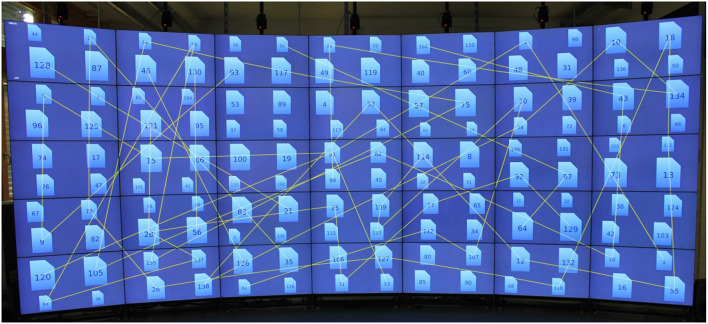
Overview Task: During the task, participants had to connect all documents ensuing from the documents' IDs. The figure shows 140 document symbols. Some of the documents are connected by lines. The lines in the figure were drawn by the authors to illustrate the point.

During the task, participants had to connect all documents ensuing from the documents' IDs, like with a Connect-the-Dots puzzle (Figure 2). However, in contrast to the focus task, the system did not notify the participants regarding task completion, so they had to decide for themselves whether they were finished or not. Similar to the focus task, there was no time constraint, and the participants did not receive any strategies prescriptions for task accomplishment. The participants could start with any ID and progress in different directions (e.g., connect 3 with 4, or 4 with 3). Similar to the focus task, the documents had fixed positions, and the participants could not move them around.

To connect two documents, the participant had to decide first what document she wants to connect (*target identification*). Next, the participant had to indicate the document to the system. The participant had to place the pointer over the document and perform the tap gesture (*target selection*). Subsequently, the participant had to find a related document to the selected one and select it as well. For instance, the participant had to select the document with the ID 3 and then connect it with the document that had the ID 4 or 2. As a result, the system will draw a line which connects the two documents. To find a related document, the participant must to look at the document IDs and decide if it is related or not related to the selected one (*sensemaking*).

#### 3.2.2. Visual Representation

The participants continued working on the data set from the focus task. Thus, at the beginning of the task, the display showed 140 document symbols. During the task, the participants added new connections between individual documents. The connections had a shape of thin yellow lines. Hence, the difficulty of the task increased with the progress, since each new connection cluttered further the working area. This design decision was made on purpose to see if increasing difficulty would affect the participants' chosen strategy. During the connection process, the system drew an additional line between a selected document and the virtual pointer.

#### 3.2.3. Interaction

To connect two documents, the participant had to select them one after another using the swipe gesture to move the cursor and the tap gesture to select the document under the cursor. The connection process could be aborted by putting the cursor in a space between documents and executing the tap gesture.

#### 3.2.4. Motivating User

To force participants to work carefully, we omit a feature for removal of existing connections.

### 3.3. Design Justifications

We designed two different tasks to observe collaborative coupling and territorial behavior. In this section, we reflected upon our design decisions and expected effects.

#### 3.3.1. Collaborative Coupling

The focus task required good skills in mathematics and physics. Mathematical questions demanded a high level of concentration (e.g., *What is 24624 divided by 6?*). In contrast, answers to physics questions did not require any calculations; the participants either knew the answers or not (e.g., *In Physics, what does STP stand for?*). Combining these two types of questions, we expected to promote transitions between loosely and tightly coupled collaborative work. For instance, lack of knowledge in the physics domain should push participants toward tightly coupled collaboration, while mental arithmetic should instead dissolve tightly coupled collaboration. We also designed the focus task in a way that allows for better spatial subdivision of the task into sub-tasks; for instance, one can split documents based on display sides.

In contrast, in the overview task, we introduced relationships between documents and, as a result, mitigated the possibility of spatial subdivision. Thus, we expected that during the focus task loosely coupled collaboration would dominate over tightly coupled collaboration, while settings of the overview task would instead result in converse user behavior. We also assumed that visual distractions caused by constant pointers' and lines' movement as well as increasing difficulty had to push participants even more toward close collaboration during the overview task.

#### 3.3.2. Territoriality

We assumed that decisions made to influence collaborative coupling should affect participants' territorial behavior as well. For example, the lack of possibility to divide the task into sub-task based on display regions in the overview task should decrease the number of territory types drastically in comparison to the focus task. Since fixed-position data withdraws an important technique for territory creation, namely grouping and our interface implemented only one explicit territory (question window), we were afraid that these circumstances would mitigate territorial behavior. To counteract this, we did not place any visual elements of the interface (apart of pointers) behind the bezels to provide a clear separation of display regions. Thus, we expected to create some pseudo-grouping - using the gestalt principle of common region (Palmer, 1992) - and to increase territoriality sensation by participants. We also utilized the highest and the lowest row of our display to investigate participants' attitude toward these critical regions and to determine what types of territories are more suitable for them.

#### 3.3.3. Smartphone Usage for Interaction

The common ways to interact with LHRDs are: (a) direct from up close using touch devices, (b) from up close or from a distance using mid-air devices (pointing or mobile), (c) from a distance using stationary devices. We did not consider stationary input devices, like mouse and keyboard, as well as workstations like laptops used for input, since they tether the user, reducing the benefits of physical navigation (Ni et al., 2006a; Ball et al., 2007). There is also a problem with mid-air pointing devices since they are not suitable for complex inputs like text input or image drawing due to lack of precision. Moreover, there are some other problems related to this type of devices. For instance, Kopper et al. identified five issues related to mid-air pointing devices for interaction: natural hand tremor, Heisenberg effect, mapping varies with distance, no parkability, no supporting surface (Kopper et al., 2008).

Although direct interaction seems to be most favorable among researchers, it is not always applicable, e.g., due to the dimension/construction of some LHRDs. Additionally, it suffers a lack of interaction at a distance. According to Ni et al. the issue of reaching distant objects is one of the main LHRD usability issues (Ni et al., 2006b). Direct interaction techniques prone mostly to this issue because users' physical abilities determine reachable areas.

On the other hand, mobile device based interaction techniques untether the user and therefore allows to interact from any distance. It also avoids most pointing devices issues and ensures access to remote display areas. Although a simple pointing device would probably perform better with our simplified tasks, it would definitively expose many drawbacks if used with real sensemaking applications.

### 3.4. Apparatus

**Figure 3:**
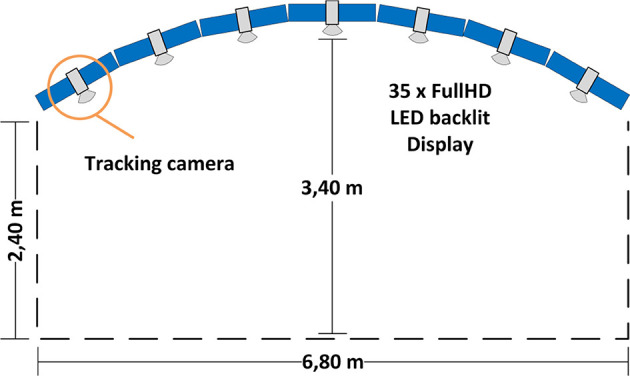
Apparatus (top view): a curved display built of 35 Full HD displays with seven tracking cameras on it that allow for tracking in front of the display within an area of around 20 square meters.

The study utilized a large, curved tiled-display (henceforth display) comprising 35 LCDs (henceforth display units) ordered through a seven (column) by five (row) grid. Each of the columns has a relative angle difference of 10 degrees along the Y-axis to adjacent columns, as such, creating a slight curvature (Figure 3). Each display unit has a bezel of fewer than 3 mm, minimizing the visual rim effect. The display units are 46” panels with a 1,080 p resolution, resulting in a total of 72 megapixels. Please note the display in question is a rigid installation, hence it could not be changed without tremendous effort. We are aware that the curvature of the display might influence user awareness. However, at such display dimensions and considering our tasks, the effect will be like a flat display of the same size. For instance, users staying together will perceive as much information regarding partner's activities as with a flat display, while staying at the sides of the display users will not be able to perceive partner's activities.

We used an array of seven infrared cameras (ARTTrack, Figure 3) to track the users' heads through head-worn helmets. We could track the helmets within an area of around 20 square meters directly in front of the display. For interaction purposes, we utilized two smartphones with similar performance characteristics. The smartphones ran an application to control pointer properties and position on the display. The application captured swipe and tap gestures and conveyed that data to the main application. The main application, in turn, made use of the data in different ways depending on its internal state. For instance, the tap gesture either opened a question or selected an answer in the focus task, while in the overview task, it either started or finished the process of connection drawing. The swipe gesture was translated into pointer movements, or allowed to choose an answer in case of an opened question window. Though we did not measure latency on the smartphone, the system allowed for smooth and highly responsive interaction with the wall display content.

The experiment supervisor, who was sitting outside the tracking area, observed and logged the following participant activities: verbal communication, transitions from loosely coupled work to tightly coupled work and vice versa, and other salient behaviors and activities. Additionally, we made video recordings for in-depth analysis.

### 3.5. Participants and Ethics

The experiment took place with 12 groups of two participants each, aged between 18 and 39 years (M = 25.08; *SD* = 4.90), with normal or corrected-to-normal vision. There were 11 female participants and 13 male participants. Random assignment of participants to groups yielded three types of group configurations: three male groups, two female groups, and seven mixed groups.

Seven groups contained participants that did not know each other and had never worked together. Four groups contained participants that did know each other and had worked together on some projects in the past. One group contained participants that did know each other, yet had never collaborated before.

With regards to language, seven groups contained participants with the same day-to-day language and five groups that contained participants with different day-to-day languages. All groups with different language backgrounds communicated in English.

The participants had an average level of computer games experience (M = 3.67; *SD* = 1.62) and mobile games experience (M = 3.08; *SD* = 1.35). Half of the participants had never seen a large, high-resolution display before (12 participants 50%). Other participants had either already seen that kind of display (9 participants 37.5%), or even worked with it (3 participants 12.5%).

All participants had an academic background (students or research associates). Each participant was paid 15 Euros for taking part in the experiment. Each participant took part only once in the experiment. We did not require approval for the study as per local legislation.

### 3.6. Procedure, Data

**Figure 4:**
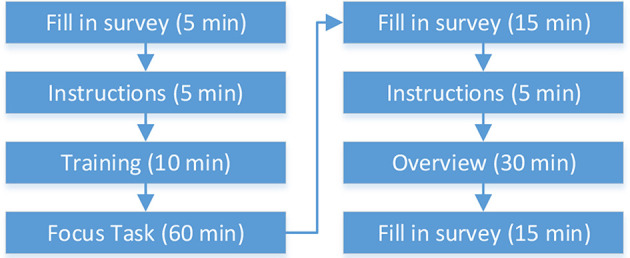
User study procedure: numbers in brackets show how much time in minutes participants required on average for individual phases.

The procedure comprised eight steps (Figure 4). First, the participants had to fill in a survey (*Personal Survey*) that encompassed questions regarding age, sex, first language, eyesight, wall-sized display experience, PC games experience, mobile game experience, height, and partner (co-user).

Next, the supervisor instructed the participants about the experiment procedure, explained the individual tasks, and how to interact with the application using the provided input device (*Instructions*). The supervisor also stressed the importance of teamwork, noted that it is up to participants how they would approach the tasks, and asked the participants to be as fast and as precise as possible. Finally, participants were instructed to stay in the tracking area. The area was bounded by the display in the front, by a thick white line on the floor in the back, and by walls on the sides.

The briefing was followed up by the training phase (*Training*). Participants were motivated to try out the interaction devices, to solve some sample tasks, and to ask questions. There was no time constraint for this stage. The transition to the *Focus task* took place after both participants indicated their readiness.

After the completion of the *Focus task*, the participants were asked to fill in a *Questionnaire*. The questionnaire encompassed multiple questions about different aspects of the study: interface, large display, the input device, and collaboration. The participants filled in the questionnaire twice once after each task. We derived the questions from the NASA TLX questionnaire (Hart and Staveland, 1988) (e.g., How mentally demanding was the task in general? Or How mentally demanding was it to work with such amount of data?). The participants answered the questions using a 7-point Likert scale.

Next, the *Overview task* took place followed by the questionnaire at the end.

During the study, we gathered quantitative and qualitative data. Quantitative data encompasses participants' position in front of the display (logged every 100 ms), pointer positions (logged on every position change), and task-related system events like the opening of a question, answering of a question, and connection of documents. Qualitative data encompasses surveys, field notes, and video recordings. In total, we captured 877 min of video/audio data. Because of a defective camera, we recorded two experiment runs only partially. That led to 65 min of lost video/audio data. Fortunately, we could acquire missing information from field notes.

We analyzed and visualized both qualitative and quantitative data to obtain the results presented in the paper. Video recordings were analyzed multiple times. During the video analysis process, we consulted the field notes and quantitative data.

## 4. Results and Discussion

In this section, we compare the results for both task conditions. We start with general information and feedback. Next, we look at different manifestations of collaborative coupling, and at the end, we look at the territorial behavior of the participants. For a better understanding of the results, we tagged groups with prior collaboration experience using the *pce* = *prior collaboration experience* subscript (e.g., for group 7 we write 7_*pce*_) whenever we mentioned specific group numbers.

### 4.1. General Feedback

The participants found the focus task more mentally demanding and more frustrating than the overview task (Table 1). Moreover, *questions answering* and *work with the given amount of data* were assessed as most mentally demanding and frustrating. In comparison, the *collaboration process* showed a rather low mental demand and did not frustrate the participants. Furthermore, the participants perceived it as successful.

**Table 1:** Assessment of different aspects of the focus and overview tasks by the participants: Mental Demand (1 - low demand, 7 - high demand); Performance (1 - Perfect, 7 - Failure); Effort (1 - low, 7 - high); Frustration (1 - low, 7 - high).

**Task 1**				
	**Mental demand**	**Performance**	**Effort**	**Frustration**
In general	M = 5.50 *SD* = 1.15	M = 3.54 *SD* = 0.81	M = 5.16 *SD* = 0.98	M = 4.46 *SD* = 1.44
Data amount	M = 4.25 *SD* = 1.53	M = 3.33 *SD* = 0.94	M = 4.20 *SD* = 1.32	M = 3.75 *SD* = 1.59
Collaboration	M = 2.29 *SD* = 1.17	M = 2.79 *SD* = 1.22	M = 3.12 *SD* = 1.45	M = 2.12 *SD* = 1.45
Questions	M = 5.33 *SD* = 1.34	M = 4.00 *SD* = 1.00	M = 5.04 *SD* = 0.79	M = 4.54 *SD* = 1.55
**Task 2**				
In general	M = 4.08 *SD* = 1.91	M = 2.25 *SD* = 1.30	M = 4.79 *SD* = 1.38	M = 3.54 *SD* = 1.80
Data amount	M = 4.67 *SD* = 1.65	M = 2.25 *SD* = 1.30	M = 4.79 *SD* = 1.38	M = 3.58 *SD* = 1.75
Collaboration	M = 2.58 *SD* = 1.68	M = 2.16 *SD* = 1.34	M = 3.46 *SD* = 1.50	M = 2.42 *SD* = 1.47

*The questions were regarding the task in general (How mentally demanding was the task? How successful was the participant? How hard did the participant work? How frustrated was the participant?), data amount (e.g., How mentally demanding was it to work with that amount of data?), collaboration (e.g., How mentally demanding was collaboration?), and questions (e.g., How mentally demanding was answering of the questions?)*.

We also asked the participants if the *interaction device and techniques were satisfying, easy to understand, and easy to master*. For both tasks, participants found the interaction device highly or rather satisfying (task 1: M = 6.08, *SD* = 0.70; task 2: M = 5.58, *SD* = 1.35), very comprehensible (task 1: M = 6.79, *SD* = 0.40; task 2: M = 6.67, *SD* = 0.55), and very easy to use (task 1: M = 6.79, *SD* = 0.40; task 2: M = 6.25, *SD* = 1.13). Although users appreciated the possibility to adjust pointer properties, they rarely made use of it. During the focus task only one user per group (in 8 of 12 groups) changed pointer properties.

### 4.2. Collaborative Coupling

As mentioned above, the process of collaborative coupling can be expressed, among others, by collaboration tightness, coupling styles, user roles, and task subdivision strategies. In this section, we look into the effects of task conditions on different manifestations of collaborative coupling.

#### 4.2.1. Collaboration Tightness

**Figure 5:**
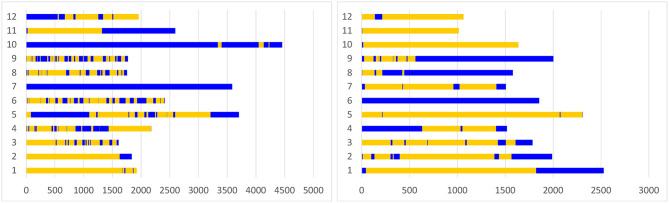
Periods of loosely coupled and tightly coupled work during the focus task (left) and the overview task (right): the Y-axis represents individual groups. The X-axis shows durations of loosely (yellow) and tightly (blue) coupled work periods in seconds, as well as time points of transitions. Groups 4, 7, 9, 10 had prior collaboration experience.

**Figure 6:**
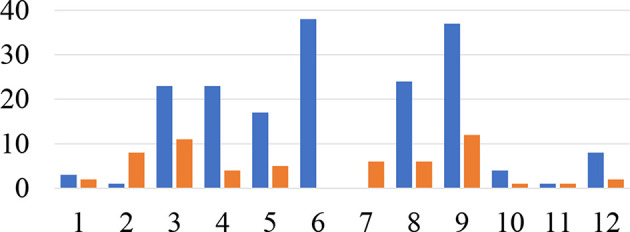
Number of transitions per group: the X-axis represents individual groups. The Y-axis shows the number of transitions (blue - focus task, orange - overview task). Groups 4, 7, 9, 10 had prior collaboration experience.

Overall, the participants spent equal amount of time working loosely coupled (Σ = 14,702 *sec*; *M* = 1225.16; *SD* = 601.46) and tightly coupled (Σ = 1,257 *sec*; *M* = 1257.67; *SD* = 1350.67) during the focus task. Groups 7_*pce*_ and 10_*pce*_ worked predominantly tightly coupled, while groups 1 and 2 worked predominantly loosely coupled. Other eight groups frequently switched between loosely and tightly coupled collaboration (see Figure 5), thus exposing a typical mixed-focus collaboration workflow. During the overview tasks, the participants made transitions less frequently (see Figures 5, 6), and spent more time working loosely coupled (Σ = 13256 *sec*; *M* = 1104.67; *SD* = 670.14) than tightly coupled (Σ = 7532 *sec*; *M* = 627.67; *SD* = 647.29).

**Figure 7:**
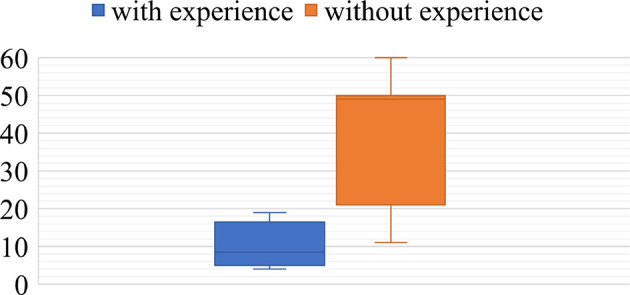
Number of CloseTask-events for groups with and without previous mutual collaboration experience.

Additionally, we detected a significant difference in collaboration tightness for groups with and without the previous mutual experience of cooperative work during the focus task. The groups with previous mutual experience discussed more frequently individual questions. The observation was confirmed by quantitative data as well. We utilized CloseTask-Event as an indicator of intra-group behavior. The event was fired by the system each time the participant closed a document without answering the question. The result revealed that groups with previous mutual experience of cooperative work left significantly fewer questions for later (M = 10.00 *SD* = 6,37) in comparison to the groups where participants have never worked together (M = 39.71 *SD* = 17.75) (Mann-Whitney *U*-test, *p* = 0.018). Figure 7 depicts the difference. Most CloseTask-Events (84) exposed the group where participants knew each other, yet have never worked together.

The findings show that task conditions have a significant effect on collaborative tightness. On the one hand, during tasks that require an advanced level of expertise in a task-related domain (like in the focus task) users can experience a lack of knowledge or uncertainty. In this case, three possible reactions were possible: the participant would guess and answer; the participant would ask for help; the participant would close the question without answering it. As a result, a particular group type will expose firm mixed-focus collaboration behavior. On the other hand, tasks that do not require any particular knowledge, but diligence only, will instead proceed in a loosely coupled manner. That means that putting users in a collaborative environment does not automatically cause collaboration. For instance, we observed extreme cases where participants processed only a few last documents mutually.

Consequently, one has to consider and support both types of collaborative coupling when designing a groupware system for sensemaking or any other complex task. Considering the finding, we could improve our system, allowing to display of the document content on the smartphone. That would reduce visual clutter on the shared display, causing less distraction for groups working loosely. Groups working tightly together could still open documents on the large display or share the smartphone display. A better solution, however, is to utilize a system that can automatically recognize the current state of collaboration tightness and adjust interaction and visualization modalities appropriately. Sigitov et al. conducted some initial work in this direction (Sigitov et al., 2018a).

#### 4.2.2. Coupling Styles

We looked for collaborative coupling styles during the study and while analyzing video recordings. We utilized the coding schemes of coupling presented in Isenberg et al. (2012) and Tang et al. (2006) as templates for our observations and combined them to a joined set. Since the schemata are problem-based, we defined the problems for our tasks as follows: answer a question – for the focus task; find a match for document A – for the overview task. Our interface did not allow for coupling style “*same problem, different areas”* (Isenberg et al., 2012) during the focus task, as well as “*same information, different views”* and “*same problem, different informations”* (Isenberg et al., 2012) during both tasks. Thus, we excluded these codes from the set.

At the beginning of each task, a short coordination phase took place [similar to *discussion* style in Isenberg et al. (2012)] where participants discussed how they should approach the task. Only two groups in the focus task and one group in the overview task went for tightly coupled collaboration where participants processed questions or connected documents mutually (“same problem, same area” style Tang et al., 2006; Isenberg et al., 2012).

The analysis yielded matches for each coupling style in the set (see Table 2). The most common style was “different problems” (Tang et al., 2006; Isenberg et al., 2012). Additionally, during the overview task, 6 of 12 groups exposed an interesting coupling style periodically. It is, though, even for humans hard to detect. First, the participants were working loosely. Then at some point, one participant asked the partner for help (e.g., “*I am looking for X, if you see it, then tell me”*). That caused the transition to a new coupling. Starting from this point, participants worked both loosely and tightly coupled, since they tried to solve not only their problem but their partner's problem as well. Liu et al. observed similar behavior (Liu et al., 2016).

**Table 2:** Collaborative coupling styles observed by Tang et al. (2006), by Isenberg et al. (2012), and in our study (• = observed, ex = excluded).

**Styles**	**Tang et al. (2006)**	**Isenberg et al. (2012)**	**Our study task 1**	**Our study task 2**
Discussion		•	•	•
Same problem,	•	•	•	•
same area				
View engaged	•	•	•	•
Disengaged	•	•	•	•
Different problems	•	•	•	•
Same general problem		•	•	•
Same problem,	•	•	ex	•
different areas				
Same information,		•	ex	ex
different views				
Same problem,		•	ex	ex
different informations				
One working,	•		•	•
another viewing				
*Multiple problems*,				•
*different areas*				

*The style multiple problems, different areas have not been observed in previous studies*.

Our result, as well as results from previous research on collaborative coupling (e.g., Tang et al., 2006; Isenberg et al., 2012), shows that environment and task characteristics (e.g., fixed-position data) might affect what coupling styles users will (be able to) employ. In our study, however, the task conditions had a marginal effect on coupling styles, since we could observe almost all of them in both conditions. However, we suggest the investigation of coupling styles for each specific task, task setting, and system type. For instance, what will happen if more than two persons collaborate? *Will new styles emerge, or some known styles vanish? Will two discussing people distract the third one who is currently working loosely? If so, should we incorporate mechanics for protection or is distraction level negligible?*

During the analysis, we found that the schemes for coupling styles constructed through users' visual attention and level of verbal communication are not able to express coupling in-depth. For instance, the view engaged coupling style described in (Isenberg et al., 2012) and (Tang et al., 2006) is typical for a partner-partner relationship. In our study, however, we observed view engaged coupling in the context of leader-assistant relationship as well. If the leader was the view-engaged user, then the assistant was the one who interacted, and the leader commented/gave instructions. In case the roles were distributed differently, the leader was the one who interacted and commented/gave instructions. The assistant remained still view-engaged, yet communicated rarely. Therefore, we suggest adding user roles to the coupling style classification.

#### 4.2.3. User Roles

We identified five user roles for tightly coupled work, whereby the leader and assistant roles were observed during the focus task only while the finder and executor roles were observed during the overview task only:

- *Partner*: both users have equal rights. This role was common for strategy discussions, situations where both participants did not know the right answer, and by opening the questions. “Do you agree?” and “Is it OK with you?” were phrases that often indicated the phases of partnership.
- *Leader*: the user who makes decisions and issues orders. We observed the role during the opening and answering the questions. The leader was usually the one who talked. Leaders decided what questions to open next and how to approach questions. Leaders often interacted with the system by themselves, though often delegated this task to their assistant.
- *Assistant*: the user who is a counterpart of the leader. They executed orders, helped if asked, and rarely made suggestions. Often, if the leader did not delegate any tasks to the assistant for a while, the assistant would part from the leader and started to work loosely coupled.
- *Executor*: the user who connects documents during the second task. Similar to partners, we did not observe any hierarchy by executor and finder.
- *Finder*: the user who searches for a match. We observed two cases. In the first case, there was a permanent finder, who looked for a match and actively indicated (verbal, using gestures and virtual pointer) to the executor, and continued looking for the next match. In the second case, there were two finders, and the executor role was assigned dynamically (the one who could perform connection faster became an executor, the other continued searching for the next match).

The user roles we observed fit any analytics task (partner, leader, assistant), and any classification/sorting task (executor, finder). For instance, the leader-assistant roles are similar to sensemaker-forager roles described in Vogt et al. (2011), yet describe the relationship and user activities in a more general way. Previous research on user roles (e.g., Vogt et al., 2011), suggests fostering user roles in groupware, for instance, employing different interfaces, views, and filters. However, in this case, user interfaces should support the dynamic switch of user roles. During the study, we observed a frequent change of user roles by the participants. Partners became leader and assistant; leaders became assistants; executors became finders and vice versa. Groupware systems should ensure equal input possibilities for all users and the seamless transfer of territorial rights to support such dynamics. Equal input possibilities will allow users to undertake different activities without negotiating much. Coordination of actions can diminish in that case to verbal notification of intentions (e.g., “I will connect these documents” or “I will put this document in the bucket”). Settings that provide only one input device for all users will likely increase coordination costs, thus making the roles more rigid and impeding collaboration (Rogers and Lindley, 2004).

Seamless transfer of territorial rights is another important design factor. In our study, the participant who opened a document became its owner and acquired rights for interaction with it. In case the owner had the assistant role, the leader—being unable to control the document—had to instruct what answer to choose. However, such limitations might become an issue if a more sophisticated input is required. In this case, the possibility to hand over ownership rights for a document (or a territory, if talking in more general terms) will allow for more flexible collaboration flow.

#### 4.2.4. Task Subdivision Strategies

Most groups decided to subdivide the focus task into spatial regions since its design predestines to such decision. Tse et al. detected similar behavior (Tse et al., 2004). Opposite to the focus task, we assumed that the absence of the possibility for the spatial subdivision would force participants to work tightly coupled. The results, however, did not confirm the assumption. The participants split the documents by IDs (e.g., from 1 upwards to 70 and 140 downwards to 70). For both tasks, we extracted the following strategies:

- Different Documents Tightly (DDT) – The participants worked predominantly on different documents (focus task) or searched for different connections (overview task). During the focus task, the participants usually portioned the display into left and right parts, while during the overview task, the participants split the documents by ID. However, they transitioned frequently to tightly coupled work for discussion or help. In comparison with other strategies, the participants left fewer documents for later during the focus task.
- Different Documents Loosely (DDL) – Same as DDT, however, the participants transitioned rarely from loosely to tightly coupled work. Mostly, these transitions took place at the end of the task (e.g., discussion of a few remaining questions or connection of a few remaining documents).
- Same Document Tightly (SDT) – The participants worked together on one question at a time (focus task) or looked for the same connection (overview task). They interacted alternately with the system and exposed rarely or no transitions to loosely-coupled work.

**Figure 8:**
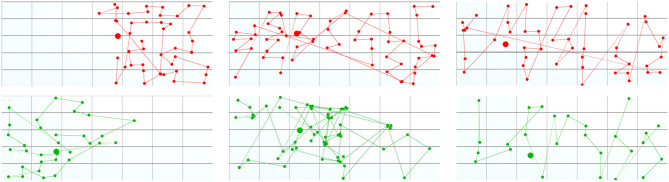
Task subdivision strategies during the focus task: **(Left)** *Different Documents Tightly*, **(Middle)** *Different Documents Loosely*, **(Right)** *Single Document Tightly*. The dots visualize positions of the cursors (top – participant 1, bottom – participant 2) during OpenTask-Events. Each line connects two consecutive events. The participants who adopted the DDT strategy worked primarily in different display regions. Though they helped each other if needed and left fewer documents for later. As a result, we can see a clear cut between the two areas. The participants who adopted the DDL strategy started similarly in different display regions, communicated, however, not much and left many documents for later. Subsequently, after the participant met in the middle of the display, they switched sides and continued to work loosely-coupled. Finally, the participants who adopted the SDT strategy worked tightly-coupled and opened documents alternately. As a result, the visualizations of the OpenTask-Events of both participants complement each other.

**Figure 9:**
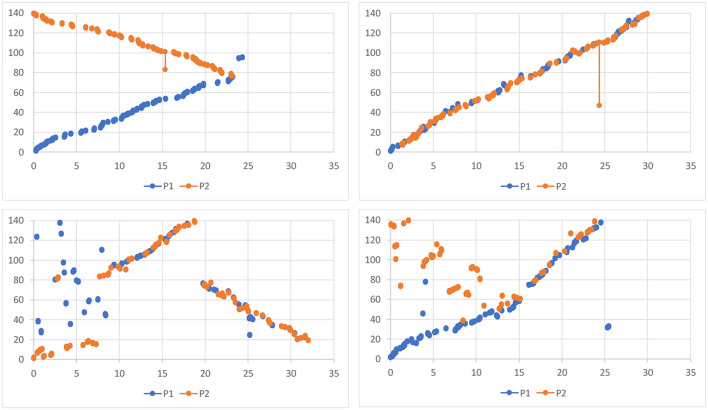
Task subdivision strategies during the overview task: (top-left) *Different Documents Loosely* – the participants started with two different IDs and worked loosely until the end; (top-right) *Single Document Tightly* – participants worked at one connection at a time; (bottom-left) *Different Documents Tightly* – although the plot is similar to one showing the *Single Document Tightly* strategy, the participants worked on two different connections at a time, yet very tightly; (bottom-right) participants started with the *Different Documents Loosely* strategy, switched, however, to the *Single Document Tightly* in the middle of the task. The Y-axis represents document IDs (from 1 to 140). The X-axis is a timeline (from 0 to 35 min). Every two dots with a line in between (blue – participant 1, orange – participant 2) visualize what documents the participants connected at what time. The more significant the difference between IDs of two connected documents, the longer is a line.

Table 3 summarizes while Figures 8, 9 exemplify based on log data different strategies for tasks subdivision the groups applied.

**Table 3:** Task processing strategies (the digit in the brackets indicates the number of groups that exposed the strategy, though did not use it as a dominant strategy): during the focus task three groups exposed the *Different Documents Tightly* strategy predominantly, seven the *Different Documents Loosely* strategy, and two the *Same Document Tightly* strategy.

**Strategy**	**# of occurrences (focus task/overview task)**
DDT	3 / 1 + (3)
DDL	7 / 7 + (1)
SDT	2 / 1 + (2)

*During the overview task, one group exposed the Different Documents Tightly strategy predominantly and three groups partially; seven groups exposed the Different Documents Loosely strategy predominantly and one group partially; and one group exposed the Same Document Tightly strategy predominantly and two groups partially*.

Our results differ from previous research (e.g., Tang et al., 2006), where participants worked mostly in a tightly coupled manner. As discussed above, previous experience of mutual collaboration seems to have an impact. However, our observations suggest that other factors might be in play as well. For instance, we noticed that participants who worked predominantly loosely coupled at the beginning of tasks tended to work more tightly at the end of tasks. Since the tasks at the end were more challenging as at the beginning (e.g., it was more challenging to find remained connections because of visual clutter), we assume that the easiness of the task should have an influence, as well as the size of the display in conjunction with fixed-position data. This configuration drove apart many participants during the focus task because of their strategy to partition the task spatially (left and right side). In comparison, the displays used by Tang et al. did not allow for long distances between participants (Tang et al., 2006), while the setting utilized by Jakobsen et al. did not contain fixed-position data (Jakobsen and HornbÆk, 2014). Moreover, we observed that when participants stood close together, they tended more to work tightly.

### 4.3. Territoriality

We observed territorial behavior by participants both on and in front of the display during both tasks. It was more salient during the focus task than during the overview task, probably because of the possibility for better task subdivision in spatial regions. The observations revealed as well that participants made excessive use of bezels to define territories. Andrews et al. and Sigitov et al. observed the similar behavior (Andrews et al., 2010; Sigitov et al., 2018b).

#### 4.3.1. Territories

In total, we observed eight types of territories during the focus task. We describe these types concerning visual elements and spatial positions on or in front of the display.

- *Personal* (similar to Scott et al., 2004) and *Personal-Shared*: represented by a question window. One instance of this territory type occupied exactly one display unit. The system reserved this area for the participant who opened the question. Therefore, no attempts were made by co-workers to operate in this area. The territory expressed multiple semantics during the task. In the case of loosely-coupled work, it was a *personal territory*. In the case of tightly-coupled work, it was a *personal-shared territory*. We do not call it *group territory* since only one participant had control over it. In contrast to personal territories on tabletops (Scott et al., 2004) and multitouch vertical displays (Jakobsen and HornbÆk, 2014), personal territories in our study were not always in direct proximity to their owner.
- *Personal-Reserved*: a display unit with a pointer inside. In case of loosely-coupled work, the participants perceived this real estate of the display unit with a pointer inside as personal territory. Co-workers made no attempts to open a question on that display unit. Participants, however, felt free to trespass this territory with their pointer.
- *Personal-Surrounding*: a column in which the participant is working. We observed that participants did not work in this territory if they could work elsewhere. Participants were more respectful of this territory in case the owner stood directly in front of it.
- *Temporary Abandoned*: sometimes, due to a transition from loosely-coupled to a tightly-coupled work style, personal territories became abandoned for a while. Such territories do not provide any drawbacks in the case of two collaborators. However, it might have a negative effect if more than two users work together.
- *Group* (similar to Scott et al., 2004): the entire display represented a group territory during the overview task. The participants worked loosely and tightly coupled within this territory. In case of tightly coupled work, the territory had region masters. Regions had a fuzzy vertical border somewhere in the middle of the display. Region masters looked for documents in their regions first.
- *Storage* (similar to Scott et al., 2004): storage territories were represented by display units that do not contain participants' pointers and do contain unprocessed questions.
- *In-between*: physical space between the participant and the area on the display the participant was working. The participants were very respectful of this territory and tried not to overstep it. Often the participants indicated their intention to trespass the territory through body signals, like starting moving movement, but not moving. If the participant saw that the partner received the signal (and showed no objections/or even approved the intention), the participant trespassed the territory.

Although the territorial behavior was not particularly salient—probably due to the employed indirect interaction technique (Ha et al., 2006)—we could observe that the participants were highly sensitive to three territory types: *personal territory, personal-reserved*, and *in-between territory*. Since the interface did not allow for interaction on a display unit occupied by a question window, the participants did not even try to work on display units on those their partners were working. Such display units were indicated either by a question window (*personal territory*) or by a pointer (*personal-reserved territory*). Thus, we conclude that explicit territories–territories implemented within a system–are less sensitive to interaction devices and techniques, and possess potential to lessen coordination workload.

**Figure 10:**
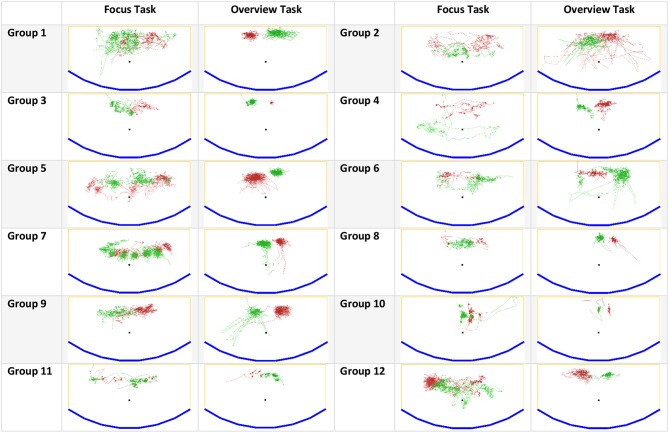
Participants' movements during the focus and overview tasks: blue – the wall-sized display, yellow – the boundaries of tracking/working area, green and red – participants' movements. Groups 4, 7, 9, 10 had prior collaboration experience.

We also could observe the effect of fixed-position data on territoriality and user interaction. Fixed-position data in our scenario required much more physical navigation (see Figure 10) in the form of full-body movements (prevailed in the focus task) or head movements (prevailed in the overview task) since the participants had to process data in all display regions. Moreover, participants could not set up a permanent territorial environment since they could not move data assets. Instead, they roamed in front of the display and used its physical features to define territories. Thus, territoriality was extremely dynamic in comparison to studies with floating data items (e.g., Scott et al., 2004; Jakobsen and HornbÆk, 2014).

#### 4.3.2. Critical Regions

One unique aspect of applications with spatial data is that users must work on every display region that contains data. That circumstance might raise an issue of critical regions. For instance, Azad et al. and Jakobsen et al. observed that users avoid lower regions of the display, probably because it was uncomfortable to interact with them (Azad et al., 2012; Jakobsen and HornbÆk, 2014). In our setup, we utilized a wall-display that includes very high display regions (over 3.0 meters) as well as low display regions (20 centimeters from the ground). We were curious to find out the participants' attitude toward these regions, so we placed data in the highest and the lowest row as well to force participants' activities within.

At the end of each task, we asked participants if it was comfortable to work in these regions. Only four participants (after the focus task) and two participants (after the overview task) found the lowest row uncomfortable. The participants named decreased legibility as the reason. Significantly more participants felt uncomfortable toward the highest row: 12 participants out of 24 (after the focus task) and 8 participants (after the overview task). The reason was the high physical demand as participants must hold their head in an abnormal position for a while. Some participants stated at the end that physical demand decreased in the overview task since they only had to glance at the highest row and not gaze at it for a long time. Hence, we suggest using high display regions for explicit territories that do not require users' attention for a long time, e.g., storage territories.

## 5. Conclusion

We conducted an extensive study that targeted different task conditions of co-located collaboration on a large, tiled-display using smartphones for interaction. Observing participants, we focused on collaborative coupling and territorial behavior, since–in our opinion—there is still a lack of understanding of these phenomena in the context of wall-sized display.

We investigated collaborative coupling regarding collaboration tightness, coupling styles, user roles, and task subdivision strategies. The study confirmed some findings from the previous research and revealed new user roles and a new coupling style that lies on edge between loosely coupled and tightly coupled styles. Both findings are datatype independent and might be generalizable to applications with not fixed-position data as well. The findings are important for the design of groupware systems and user interfaces. Ideally, the system should be intelligent enough to recognize users work style and appropriately adjust the interface (e.g., Sigitov et al., 2018a). For that, researchers have to extract, categorize, and describe patterns of user and group behavior (e.g., in the form of coupling styles or user roles) in a way the system based on sensors' data could recognize them.

The study also revealed that putting users into a collaborative environment does not automatically cause close collaboration. More likely, users will search for task subdivision possibilities (e.g., spatial or logical) and process the sub-tasks in parallel. However, the tightness of collaboration depends on other factors. In our study, for instance, we detected that most groups with previous mutual collaborative experience worked more tightly in comparison to other groups, while lack of knowledge and uncertainty amplified the effect. As a result, we suggest other factors for future investigation: easiness of the task, and size of a shared display in conjunction with fixed-position data, as well as investigation of previous collaboration experience on group coupling/behavior.

We observed different coupling styles than revealed in previous research. However, we have to note that definitions of these coupling styles do not manifest the essence of coupling in enough granularity. Hence, we suggest utilizing user roles to extend the coupling description.

Regarding territoriality, we observed some mitigation of territorial sensitivity, probably caused by the employed indirect interaction technique (Ha et al., 2006). However, we could also detect that participants remained very sensitive to three territory types: *personal territory, personal-reserved*, and *in-between territory*.

The physical territory between the participant and the working area on display increased coordination workload. Since the tracking area limited the participants, and most of them stayed at the posterior border of it, there was no way to circuit the partner from the back. Thus, the participants had to coordinate their work by employing expressions of intentions and short agreements. Therefore, we suggest designing workspaces in a way that do not inhibit participants from changing their locations, especially if using handheld interaction devices.

We also suggest further investigation of another territory type never mentioned in the literature before, namely *temporary abandoned territory*. In this study, the participants had never noticed this territory type since they emerge only if one participant left the personal territory for tightly coupled work within another territory. We assume, however, that this kind of territory might have an adverse effect if the number of co-located participants increases.

Finally, we found that the participants did not perceive all display regions comfortable. The highest row of display units caused physical stress by half of the participants once they had to gaze at it for a while. In contrast, the lowest row did not cause any problems, thus increasing valuable display real estate. It has been shown though that with touch displays (e.g., Jakobsen and HornbÆk, 2014), users' frustration would instead increase, since they would have to bow and crouch in front of the display to interact within low regions. Sure enough, the task itself plays an important role here. For instance, Von Zadow et al. showed that in the gaming context users might have a positive attitude toward the required physical effort (von Zadow et al., 2016).

We assume that changing the number of participants or collaboration type will likely influence the results as well. Thus, in the future, we will increase the number of participants. Moreover, we are going to investigate co-located collaboration on a wall-sized display in the context of the concrete task, namely game level design, which is also based heavily on fixed-position data.

## Ethics Statement

This study was carried out with written informed consent from all subjects. All subjects gave written informed consent in accordance with the Declaration of Helsinki.

## Author Contributions

AS conducted the study, analyzed the study results, and wrote the first draft of the manuscript. All authors contributed conception and design of the study, manuscript revision, read, and approved the submitted version.

### Conflict of Interest

The authors declare that the research was conducted in the absence of any commercial or financial relationships that could be construed as a potential conflict of interest.
