# Supplementary material for: Task Dependent Group Coupling and Territorial Behavior on Large Tiled Displays
Source: Front Robot AI. 2019 Nov 26;6:128. doi: 10.3389/frobt.2019.00128 (PMC7805936; doi:10.3389/frobt.2019.00128)
Supplement: Supplementary file 1 [file Data_Sheet_1.PDF]

Questions and answers used during the Focus task

1. If 2468 is subtracted from 8642, what is left?
    a. 6174
    b. 6176
    c. 6214
    d. 6274
2. What is the next number in the sequence 7, 18, 62, 238?
    a. 812
    b. 872
    c. 942
    d. 972
3. What results when the cube of 4 is added to half of 8?
    a. 6
    b. 20
    c. 68
    d. 260
4. If a winning candidate secured 55% of the 12000 votes cast, how many voted for him?
    a. 6000
    b. 6400
    c. 6600
    d. 7200
5. What is 884 divided by 26?
    a. 28
    b. 32
    c. 34
    d. 38
6. What is the only prime number between 98 and 102?
    a. 99
    b. 100
    c. 101
    d. 102
7. How long will it take a car travelling at 45mph to cover 150 miles?
    a. 2 hours 50 minutes
    b. 3 hours 10 minutes
    c. 3 hours 20 minutes
    d. 3 hours 30 minutes
8. 220 books were sold at a price of £11 each. How much money was taken?
    a. 2140
    b. 2220
    c. 2222
    d. 2420
9. In a class of 32 children, 12 are boys. What percentage does this represent?
    a. 30
    b. 33
    c. 37.5
    d. 40
10. In a bag of 28 sweets, 7 of them were lemon flavour. What percentage were not lemon?
    a. 60

    b. 66
    c. 70
    d. 75
11. Which is the smallest out of two thirds, 60%, 0.65 or seven elevenths?
    a. 2/3
    b. 60%
    c. 0.65
    d. 7/11
12. What is the next number in the series 4, 17, 160, 1733 ….?
    a. 18,760
    b. 19,036
    c. 21,364
    d. 22,030
13. What is 232 multiplied by 11?
    a. 2,332
    b. 2,442
    c. 2,552
    d. 2,662
14. If 27 items are bought at a price of €2.45 each, what is the total bill?
    a. 66
    b. 69
    c. 70
    d. 72
15. What is the cube root of 1,331?
    a. 9
    b. 11
    c. 13
    d. 17
16. What is the area of a triangle whose height is 35cm and whose base is 32cm?
    a. 280 sq cm
    b. 360 sq cm
    c. 560 sq cm
    d. 1120 sq cm
17. How many seconds are there in 28 minutes?
    a. 1,680
    b. 1,684
    c. 1,690
    d. 1,696
18. What is 3,844 divided by 16?
    a. 211
    b. 240
    c. 280
    d. 291
19. If a plane covers 375km in 45 minutes, how far will it travel in 6 hours?
    a. 2,450km
    b. 2,750km
    c. 3,000km
    d. 3,200km
20. If £350 interest was received at a rate of 2.8%, how much was invested?

a. £7,500
b. £11,000
c. £12,500
d. £14,500

21. What is 27,764 subtracted from 73,351?
    a. 44,587
    b. 45,587
    c. 45,877
    d. 45,887
22. What is the square root of 784?
    a. 22
    b. 24
    c. 26
    d. 28
23. What results when the cube of 6 is added to the square root of 361?
    a. 53
    b. 55
    c. 233
    d. 235
24. What is the cost of 18kg of plums at a price of 23.5 pence per kg?
    a. 4.13
    b. 4.17
    c. 4.23
    d. 4.27
25. What is the next number in the series 2, 3, 8, 63 ….?
    a. 125
    b. 248
    c. 2848
    d. 3968
26. What is the sum of the numbers between 31 and 36 (inclusive)?
    a. 201
    b. 202
    c. 203
    d. 204
27. If two sides of a right-angled triangle are 5cm and 12cm long, how long is the third side?
    a. 6cm
    b. 9cm
    c. 13cm
    d. 17cm
28. What is the quarts in a gallon multiplied by the acres in a square mile?
    a. 256
    b. 384
    c. 2560
    d. 3840
29. What is $5^2 + 13^2 + 15^2$?
    a. 369
    b. 399
    c. 419
    d. 439

30. If a bakers dozen of items costs £3.51, what does each item cost individually?
    a. 19 pence
    b. 27 pence
    c. 29 pence
    d. 31 pence
31. What is the next number in the series 3, 12, 102 ....?
    a. 304
    b. 408
    c. 622
    d. 1002
32. A 3 hour conference uses 6 speakers. How much time will each one get on average?
    a. 15 minutes
    b. 20 minutes
    c. 30 minutes
    d. 45 minutes
33. A car costs a dealer $6,775 to buy and he sells it for $10,350. What profit is made?
    a. $3,355
    b. $3,575
    c. $3,735
    d. $3,755
34. How much is a 15% service charge on top of a bill of £84.40?
    a. 12.06
    b. 12.6
    c. 12.61
    d. 12.66
35. When will someone born in 1978 celebrate their 53rd birthday?
    a. 2011
    b. 2021
    c. 2031
    d. 2041
36. How many 250cc glasses are needed to hold 17.5 litres of water?
    a. 70
    b. 72
    c. 76
    d. 80
37. How many minutes are there between mid-day on Tuesday and mid-day on Thursday?
    a. 2008
    b. 2080
    c. 2800
    d. 2880
38. Starting with 48, three sixteenths of items are sold within an hour. How many are left?
    a. 31
    b. 33
    c. 36
    d. 39
39. Three people bank amounts of £35.75, £42.75 and £63.55 respectively. What is the total?
    a. 132.05
    b. 138.35
    c. 141.55

    d. 142.05

40. What is the cost of 120 chocolate bars at 31 pence each?
    a. 31.4
    b. 37.2
    c. 39.6
    d. 42
41. An invoice must be paid exactly 30 days after 25th March - when will that be?
    a. 20th April
    b. 24th April
    c. 28th April
    d. 1st May
42. A CD holds 78 minutes worth of music. How many 3 minute tracks can be included?
    a. 19
    b. 26
    c. 29
    d. 31
43. A rugby team won 60% of its 30 games, and drew one. How many did it lose?
    a. 9
    b. 10
    c. 11
    d. 12
44. If steel wool costs £9 per yard, how many feet would £243 buy?
    a. 67
    b. 71
    c. 76
    d. 81
45. How many months of the year have less than 31 days?
    a. 3
    b. 4
    c. 5
    d. 6
46. What is nine squared, added to the square root of nine?
    a. 30
    b. 57
    c. 84
    d. 90
47. How many euros would £15 buy at an exchange rate of £1 = €1.48?
    a. 21.4
    b. 22.2
    c. 23.6
    d. 27.7
48. What percentage is two fifths?
    a. 25
    b. 35
    c. 40
    d. 45
49. What is 24,624 divided by 6?
    a. 404
    b. 4004

c. 4014
d. 4104

50. What is 9,342 subtracted from 17831?
    a. 7699
    b. 8039
    c. 8399
    d. 8489
51. What is one third of 102?
    a. 34
    b. 35
    c. 36
    d. 37
52. In Physics what is resistance to change in a state of motion called?
    a. Inertia
    b. Inflexibility
    c. Mass
    d. Solidarity
53. Which body causes incident light to separate by colour upon exiting?
    a. Lens
    b. Mirror
    c. Prism
    d. Water
54. What is created in space upon the collapse of a neutron star?
    a. Asteroids
    b. Black Hole
    c. Galaxy
    d. Solar System
55. What does "supersonic" speed exceed?
    a. The Speed of a Space Shuttle
    b. The Speed of Concorde
    c. The Speed of Light
    d. The Speed of Sound
56. What name is given to the path taken by a projectile?
    a. Ballistic
    b. Circumference
    c. Parabola
    d. Trajectory
57. What name is given to a device which measures atmospheric pressure?
    a. Ammeter
    b. Barometer
    c. Thermometer
    d. Weather Vane
58. What in Physics is the opposite of condensation?
    a. Compression
    b. Freezing
    c. Meltdown
    d. Vaporisation
59. As a general law of mechanics, what is stress directly proportional to?
    a. Density

   b. Force
   c. Pressure
   d. Strain
60. In Physics, what does STP stand for?
   a. Standard temperature and pressure
   b. Standard thermal policy
   c. Standard time and place
   d. Standard tonnage and placement
61. At what Fahrenheit temperature does water boil?
   a. 100 degrees
   b. 152 degrees
   c. 212 degrees
   d. 792 degrees
62. With which branch of Physics does the Kirchhoff Junction Rule apply?
   a. Electricity
   b. Light
   c. Sound
   d. Wave Motion
63. Which electronic components have impedance?
   a. Batteries
   b. Capacitors
   c. Resisitors
   d. Switches
64. In Physics, what is the angle called between a ray and the surface normal?
   a. Angle of Deflection
   b. Angle of Incidence
   c. Angle of Reflection
   d. Angle of Refraction
65. What is electric potential more commonly known as?
   a. Current
   b. Power
   c. Voltage
   d. Wattage
66. With what is Archimedes' Principle concerned?
   a. Air Pressure
   b. Buoyancy
   c. Heat Transfer
   d. Light Refraction
67. In Physics, what are the two distinctive types of friction?
   a. Inert and Dynamic
   b. Motive and Immotive
   c. Permanent and Temporary
   d. Static and Kinetic
68. What does an anemometer measure?
   a. Relative Density
   b. Relative Humidity
   c. Viscosity
   d. Wind Speed
69. What temperature on the Kelvin Scale is equivalent to minus 273 Celcius?

a. Minus 173
b. Minus 273
c. Plus 100
d. Zero

70. What frequencies of sound energy exceed the human upper hearing limit?
    a. Extrasonic
    b. Supersonic
    c. Ultrasonic
    d. Uppersonic
